# Pre-existing T cell-mediated cross-reactivity to SARS-CoV-2 cannot solely be explained by prior exposure to endemic human coronaviruses

**DOI:** 10.1016/j.meegid.2021.105075

**Published:** 2021-11

**Authors:** Cedric C.S. Tan, Christopher J. Owen, Christine Y.L. Tham, Antonio Bertoletti, Lucy van Dorp, Francois Balloux

**Affiliations:** a UCL Genetics Institute, University College London, Gower Street, London WC1E 6BT, United Kingdom; b Emerging Infectious Diseases Program, Duke-NUS Medical School, 8 College Road, Singapore 169857, Singapore

## Abstract

T-cell-mediated immunity to SARS-CoV-2-derived peptides in individuals unexposed to SARS-CoV-2 has been previously reported. This pre-existing immunity was suggested to largely derive from prior exposure to ‘common cold’ endemic human coronaviruses (HCoVs). To test this, we characterised the sequence homology of SARS-CoV-2-derived T-cell epitopes reported in the literature across the full proteome of the *Coronaviridae* family. 54.8% of these epitopes had no homology to any of the HCoVs. Further, the proportion of SARS-CoV-2-derived epitopes with any level of sequence homology to the proteins encoded by any of the coronaviruses tested is well-predicted by their alignment-free phylogenetic distance to SARS-CoV-2 (*Pearson's r* = −0.958). No coronavirus in our dataset showed a significant excess of T-cell epitope homology relative to the proportion of expected random matches, given their genetic similarity to SARS-CoV-2. Our findings suggest that prior exposure to human or animal-associated coronaviruses cannot completely explain the T-cell repertoire in unexposed individuals that recognise SARS-CoV-2 cross-reactive epitopes.

## Highlights

• An extensive phylogeny of *Coronaviridae* was reconstructed for all core genes.
• Homology to published epitopes was assessed bioinformatically across the family *Coronaviridae.*
• Endemic coronaviruses cannot explain all SARS-CoV-2 T-cell cross-reactivity.
• Exposure to diverse antigens likely helped shape SARS-CoV-2 cross-reactive profiles.

## 1 Introduction

Severe acute respiratory coronavirus 2 (SARS-CoV-2) is a member of a large family of viruses; the *Coronaviridae*, whose members can infect a wide range of mammals and birds (Shaw et al., 2020). Human coronaviruses were first described in the 1960s (Tyrrell and Bynoe, 1965) with SARS-CoV-2 now the seventh coronavirus known to infect humans; joining the epidemic human coronaviruses, SARS-CoV-1 (Ksiazek et al., 2003) and MERS-CoV (Zaki et al., 2012), and the four species of endemic human coronaviruses (HCoVs). Human endemic coronaviruses are associated with mostly mild upper respiratory infections – ‘common colds’ – and include *Coronaviridae* of the *Alphacoronavirus* genera 229E and NL63 and members of the *Betacoronavirus* genera OC43 and HKU1 (Su et al., 2016) to which MERS-CoV, SARS-CoV-1 and SARS-CoV-2 also belong. Both SARS-CoV-1 and SARS-CoV-2 fall into a subgenus of the *Betacoronavirus* named the *Sarbecovirus* (Boni et al., 2020), with approximately 80% identity at the nucleotide level between SARS-CoV-1 and SARS-CoV-2. All human coronaviruses are thought to be zoonotic in origin, though the exact animal reservoirs remain under debate in some cases (Ye et al., 2020).

SARS-CoV-2 is estimated to have jumped from a currently unknown animal reservoir into the human population towards the end of 2019 (van Dorp et al., 2020) giving rise to the pandemic disease Coronavirus disease 2019 (COVID-19). The symptoms associated with COVID-19 range from fully asymptomatic infections and mild disease through to severe respiratory disease with associated morbidity and mortality. Marked disparities exist in individual risk of severe COVID-19 with gender, ethnicity, metabolic health and age all identified as important determinants (Jordan et al., 2020; Wu et al., 2020; Zhou et al., 2020). Population age structures and heterogeneous burdens in nursing homes only partially explain the variation in infection fatality rates (IFRs) between countries (O'Driscoll et al., 2020). Further important contributors may include climatic variables (e.g. temperature and humidity) and associated seasonal correlates (Walker et al., 2020; Gaunt et al., 2010; Moriyama et al., 2020), the choice of non-pharmaceutical interventions put in place, and more recently vaccination coverage though with a myriad of other possibly unknown contributing factors.

In light of the wide spectrum of symptoms associated to COVID-19, several studies have probed antibody (Lv et al., 2020; Ladner et al., 2021; Ng et al., 2020) or T-cell responses (Mateus et al., 2020; Grifoni et al., 2020a; Weiskopf et al., 2020a; Le Bert et al., 2020; Nelde et al., 2020; Braun et al., 2020; Peng et al., 2020; Schulien et al., 2020; Bacher et al., 2020; Sekine et al., 2020; Steiner et al., 2020; Echeverría et al., 2021; Reynolds et al., 2020; Low et al., 2021) in samples from healthy individuals collected prior to the COVID-19 pandemic to test for the presence of pre-existing cross-reactivity to SARS-CoV-2. Collectively, these findings provide evidence for a degree of antibody and T-cell cross-reactivity in unexposed individuals in multiple regions of the world. While the source of this cross-reactivity remains poorly defined, at least some of the cross-reactive T-cell epitopes have been suggested to derive from exposure to the four endemic human coronaviruses (Mateus et al., 2020; Le Bert et al., 2020), which were circulating in most parts of the world prior to the COVID-19 pandemic (Su et al., 2016), typically in seasonal cycles (Neher et al., 2020). Further, SARS-CoV-2 cross-reactive epitopes have been identified in exposed seronegative healthcare workers contributing to abortive infections (Swadling et al., 2021). Such studies have been based, in part, on the degree of homology of detected epitopes to protein sequences found in each of the four HCoVs, though lacked consideration of many other coronaviruses which circulate widely in mammals or the degree of matching expected given the relatedness of these viruses to SARS-CoV-2. As such, the relative contribution of each of the four HCoVs to T-cell cross-reactivity patterns observed in unexposed individuals remains unclear. Notably, Peng et al. (Peng et al., 2020) did not find the presence of cross-reactivity in a cohort of 16 unexposed donors.

To date, it also remains unclear whether detected cross-immunity in unexposed individuals translates into consistently differential COVID-19 pathogenesis. The evidence for a mitigating role of recent HCoV infection on COVID-19 susceptibility and symptom severity upon infection is mixed (Sagar et al., 2020; Gombar et al., 2021). HCoV-reactive T-cells in unexposed individuals have been shown to have only low functional avidity (Bacher et al., 2020), though cohort studies suggest pre-exisiting coronavirus RNA-polymerase-specific T-cells are an important determinant of abortive rather than overt infection (Swadling et al., 2021). As such there has been speculation that cross-immunity with the ‘common cold’ endemic HCoVs may, in part, explain variation in the COVID-19 case-fatality rate in different parts of the world (Gupta and Misra, 2020; Yaqinuddin, 2020) and that the high incidence of common colds in children and adolescents has contributed to their markedly lower risk of severe disease (Ng et al., 2020). Additionally, the possible unnoticed circulation in the human population of another animal-associated coronavirus, at least in some regions of the world, cannot at this stage be formally ruled out to have contributed to regional heterogeneities in the spread and associated mortality of COVID-19.

In this study, we sought to probe the possible sources of pre-existing T-cell immunity in samples from healthy individuals predating the COVID-19 pandemic. One tractable way to determine the contribution of multiple human or animal-associated coronaviruses to T-cell cross-reactivity is to consider the amino acid sequence homology of experimentally-validated SARS-CoV-2 epitope sequences to proteins encoded by these viruses. The assumption is that viruses that have contributed significantly to cross-reactivity are likely to possess a higher than expected number of protein sequences with reasonable sequence homology to these SARS-CoV-2 epitopes. While we recognise that two epitopes sharing a low sequence homology can be cross-reactive due to structural conservation (Macdonald et al., 2009; Wucherpfennig and Strominger, 1995; Quaratino et al., 1995), the vast majority of cross-reactive epitopes share a high sequence homology (Mateus et al., 2020). That is, epitopes that share a higher sequence homology have a far higher likelihood of being cross-reactive. Therefore, sequence homology offers a good proxy for determining the initial antigen that elicited a T-cell response. We therefore analysed sequence conservation over the SARS-CoV-2 proteome across the *Coronaviridae*, which involved the construction of a core gene family-wide phylogeny. We subsequently assessed the amino acid homology to endemic HCoVs and other members of the *Coronaviridae* of 177 CD4^+^ and CD8^+^ epitopes identified in healthy unexposed individuals reported by four independent studies (Mateus et al., 2020; Le Bert et al., 2020; Nelde et al., 2020; Schulien et al., 2020).

We find that more than half of the reported epitopes (54.8%) did not have detectable homology to any of the endemic HCoVs. Further, none of the sequenced members of the *Coronaviridae* could explain a higher proportion of reported epitopes than expected by chance, given the phylogenetic similarity of their entire genome to SARS-CoV-2. Our results suggest that prior exposure to endemic coronaviruses is not the sole explanation of cross-reactivity patterns to SARS-CoV-2 in unexposed individuals. Instead, patterns of pre-existing T-cell cross-reactivity to SARS-CoV-2 seem largely in line with lifelong exposure to a diverse and heterogenous array of primarily microbial antigens. We anticipate that our findings will facilitate further characterisations of the potential sources of pre-existing T-cell immunity.

## 2 Methods

### 2.1 Data acquisition

3300 publicly available complete *Coronaviridae* assemblies were downloaded from NCBI Virus using the *taxid*: 1118 together with accompanying metadata on 08/04/2020. We also identified a further set of 41 Sarbecoviruses for inclusion that were released subsequent to January 2021. This dataset includes 12 bat and pangolin Coronavirus sequences from GISAID (Elbe and Buckland-Merrett, 2017) (acknowledged in Table S3). Sequence duplicates were identified and removed from the combined dataset using *seqkit rmdup* (Shen et al., 2016) together with those accessions with >10% of sites set to N. Accessions were later retained in the dataset only for those with a reported host of isolation. This resulted in a final dataset of 2572 assemblies with complete metadata with the latter manually cleaned to ensure consistent reporting of host and viral species.

### 2.2 Maximum Likelihood phylogeny of Coronaviridae

To reconstruct the core genomic diversity of the entire *Coronaviridae* family, we extracted the shared core genes from the representative genome assemblies across all genera. First, open reading frames (ORFs) were identified using the genome annotation tool *Prokka* v1.14.6 (Seemann, 2014). Next, the *Roary* pipeline v3.11.12 (Page et al., 2015) was used to cluster all *Coronaviridae* ORFs at a minimum amino-acid homology threshold of 30%. Sequences for the four genes ORF1ab, S, M and N were each found to cluster in a minimum of 2572 assemblies, which were then extracted, concatenated and aligned using *MAFFT* v7.453 (Katoh et al., 2002). The resulting alignment was trimmed of gaps found in 20% or more isolates and used to build a Maximum Likelihood phylogeny using *IQTree* v1.6.9 (Nguyen et al., 2015) specifying the *-fast* option. The four core genes in the trimmed concatenated alignment (12,014 bp) corresponds to 43.1% of the average length of all included WGSs (27,867 bp). We provide the curated metadata of the final 2572 viral records used in our analysis in Table S1.

As it was not possible to include an outgroup in the *Coronaviridae* concatenated-core alignment, an alignment-free analysis was used to identify the most basal genus with which to root the family Maximum Likelihood phylogeny. All *RefSeq* genome assemblies belonging to the virus order *Nidovirales* were downloaded, which contained 103 sequences accrsoss the sub-orders *Arnidovirineae*, *Cornidovirineae*, *Mesnidovirineae*, *Nanidovirineae*, *Ronidovirineae* and *Tornidovirineae*. Each assembly contained a ORF1ab CDS annotated ORF, the only gene shared by all members of the *Nidovirales* (Lauber et al., 2013), which were decomposed into 14-mer sequences using *MASH* v2.1.1 (Ondov et al., 2016). Based on pairwise Jaccard Distances of matched 14-mers between all ORF1ab sequences, a Neighbour-Joining tree was constructed to assess the genetic relationship between members of the *Nidovirales*. The genus *Deltacoronavirus* was identified to be the most basal clade of the *Coronaviridae* in the wider context of the taxonomic order and was therefore used to root the family-wide Maximum Likelihood phylogeny.

### 2.3 Sequence conservation analysis

We decomposed the SARS-CoV-2 proteome (sequences retrieved from *RefSeq*; NC_045512.2) into 9394 15-mer peptides overlapping by 14 amino acids using a custom *R* script. Such a 15-mer sliding window allows for consideration of all possible peptide strings within the SARS-CoV-2 proteome. In addition, we retrieved the sequences of 177 epitopes found to elicit a response in at least one individual unexposed to SARS-CoV-2 from Singapore (Le Bert et al., 2020), the USA (Mateus et al., 2020) and Germany (Nelde et al., 2020; Schulien et al., 2020) from published supplementary tables. The breakdown of the number of epitopes for each T-cell response type is shown in Table S4b. Translated protein sequences of all ORFs from each of the 2572 assemblies were retrieved from *Prokka* (Seemann, 2014) and used to construct a protein BLAST database. Separately, a protein BLAST database was also constructed from the protein annotations associated with the 2572 assemblies, which were downloaded using *NCBI Batch Entrez* (https://www.ncbi.nlm.nih.gov/sites/batchentrez). Subsequently, we used the BLASTP utility from *BLAST+* v2.11.0 (Camacho et al., 2009) to determine the sequence homology of the 15-mer peptides from the SARS-CoV-2 proteome and the 177 published epitopes using the two databases. Sequence homology (or percentage identity), is defined here as the percentage of amino acid/nucelotide matches between any two sequences. The resultant protein BLAST outputs were merged by retaining only the hit with the maximum percentage identity for each assembly and query combination. To include all tested alignments, we set *-num_alignments* and *-evalue* parameters to 10^9^ and 2 × 10^9^, respectively. In addition, to optimise the protein BLAST search for short sequences, *−task* was set to *blastp-short*. Lastly, only alignments involving the full length of the query sequence were considered by setting *-qcov_hsp_perc* as 99. This threshold was employed because the query sequences are short and so sequence identity would only be a meaningful measure of homology in alignments given the whole sequence. Using this BLASTP implementation, we store the sequence homology values when an alignment was produced, and return zero for cases when it was not (referred to as ‘no homology’).

### 2.4 Regression analysis

Using the merged output of the protein BLAST search querying the 177 published epitopes, we analysed the proportion of epitopes that had any homology to each virus in our dataset. To do so we additionally calculated the alignment-free genetic distance - ‘Mash distance’ - of each virus relative to SARS-CoV-2 using *MASH v2.1.1* (Ondov et al., 2016) specifying a *k*-mer size of 14. A least squares regression of the proportion of epitopes with any homology on the natural logarithm of Mash distance was performed using the *lm* function in *R*. This analysis was applied to a representative filtered dataset of all combinations of unique host and virus species requiring a unique Mash distance to SARS-CoV-2 (*n* = 365). Pearson's correlation of the two variables was also calculated using the *cor.test* function in R. The studentised residuals were calculated using the *studres* function as part of the *MASS* package v7.3–53 (Ripley et al., 2013).

### 2.5 Non-*Coronaviridae* protein BLAST

To determine if any proteome outside of the *Coronaviridae* had detectable homology to any of the 177 epitopes reported in the literature, we performed a protein BLAST analysis using the online BLASTP *suite* (https://tinyurl.com/y22o4t9z) against the non-redundant protein sequence database (accessed 7/12/2020), while excluding sequences associated with the *Coronaviridae* (taxid: 11118). Protein BLAST searches were conducted in eight batches of 20 and a ninth batch of 17 epitopes with the number of alignments performed set to 1000 per batch. After merging the outputs of the eight batches, we filtered the resultant table to exclude missing organism names, hits with descriptions containing the terms ‘synthetic’, ‘SARS’, ‘coronavirus’, or ‘cov’, or organism names labelled as ‘uncultured bacterium’. Additionally, we excluded hits to the Protein Data Bank accession 6ZGH_A, given it contains a region of the SARS-CoV-2 spike protein sequence.

## 3 Results

### 3.1 Conservation analysis across the family-wide phylogeny of *Coronaviridae*

**Fig. 1:**
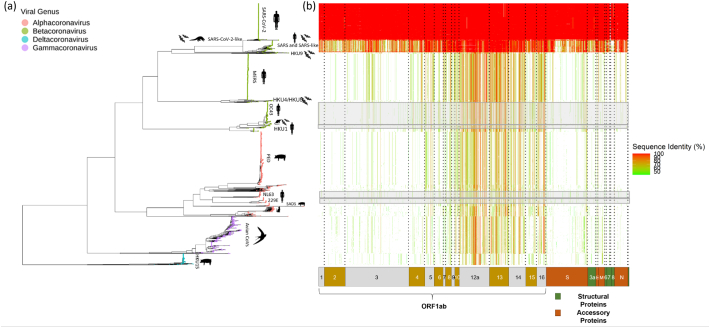
Conservation analysis of SARS-CoV-2-derived 15-mer peptides across the *Coronaviridae*. (a) Maximum likelihood phylogeny of a concatenated alignment of core genes in the *Coronaviridae* annotated by viral genera (tip colour) and highlighting major hosts (Table S1). (b) Heatmap visualising the homology of SARS-CoV-2-derived 15-mer peptide sequences across the family. Each row and column correspond to a tip on the phylogeny and a single 15-mer peptide, respectively. The fill of each cell provides the level of homology of a particular SARS-CoV-2-derived 15-mer peptide to the proteome of a single genome record as given by the colour scale at right. Grey boxes highlight the rows of the heatmap corresponding to each of the four endemic human coronaviruses. The homology threshold set to report a protein BLAST hit was 40%. Conservation analysis of SARS-CoV-2-derived 15-mer peptides across the *Coronaviridae*. (a) Maximum likelihood phylogeny of a concatenated alignment of core genes in the *Coronaviridae* annotated by viral genera (tip colour) and highlighting major hosts (Table S1). (b) Heatmap visualising the homology of SARS-CoV-2-derived 15-mer peptide sequences across the family. Each row and column correspond to a tip on the phylogeny and a single 15-mer peptide, respectively. The fill of each cell provides the level of homology of a particular SARS-CoV-2-derived 15-mer peptide to the proteome of a single genome record as given by the colour scale at right. Grey boxes highlight the rows of the heatmap corresponding to each of the four endemic human coronaviruses. The homology threshold set to report a protein BLAST hit was 40%.

To reconstruct the shared genomic diversity of the *Coronaviridae* family, we extracted a concatenated alignment of core (shared) genes (ORF1ab, S, M, N) from annotated genome assemblies of 2572 coronaviruses, isolated from human and animal hosts, and constructed a Maximum Likelihood phylogeny (Fig. 1a, Table S1). We then decomposed the SARS-CoV-2 proteome (NC_045512.2) into 15-mer peptide sequences overlapping by 14 amino acids and performed protein *BLAST* searches to determine the homology to protein sequences translated from each of the 2572 coronavirus assemblies isolated from a range of hosts (see Methods). Two sequences are said to have ‘no homology’ if a protein BLAST alignment of said sequences could not be produced. The proteome-wide homology of 15-mer peptides across the *Coronaviridae* is represented in Fig. 1b. At a 40% amino acid sequence homology cut-off, SARS-CoV-2 peptide sequences were highly conserved across the family at the C-terminal end of ORF1ab. Representations of alternative homology thresholds (66% and 80%) provide qualitatively similar patterns (Fig. S1a and b). This region of homology includes the RNA-dependent RNA polymerase (RdRp) (nsp12) and helicase (nsp13) which are known regions of high conservation across the coronaviruses, with the former frequently used as a taxonomic marker (Latinne et al., 2020).

### 3.2 Cross-reactivity profiles cannot be completely explained by exposure to endemic HCoVs

**Fig. 2:**
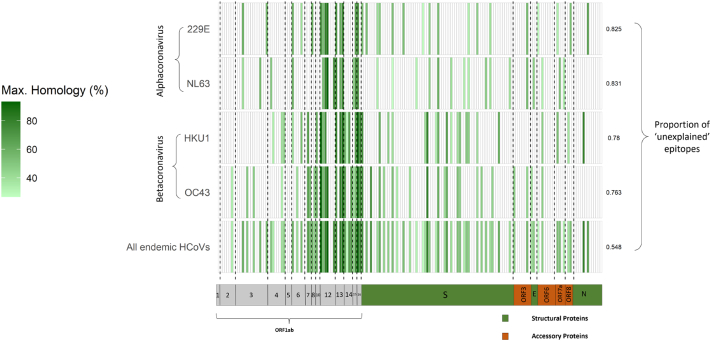
Sequence homology of deconvoluted peptides from published literature to endemic HCoVs. Heatmap visualising the maximum sequence homology of deconvoluted SARS-CoV-2-derived peptides to the each of the four endemic HCoVs (first four rows) and across all HCoVs combined (last row). The proportion of epitopes that cannot be explained by detectable homology to proteins from each species of HCoV is annotated on the right of the heatmap. Each row and column correspond to a single genome record and a single peptide, respectively. The fill of each cell provides the maximum sequence homology of a particular SARS-CoV-2-derived epitope to the proteome of all genome records for each species. This maximum sequence homology was determined by considering only all viruses isolated from a human host and with species names including the terms ‘229E’, ‘NL63’, ‘HKU1’ and ‘OC43’.

We analysed the sequence homology of 177 cross-reactive peptides found to elicit T-cell response in published work on four independent cohorts of healthy unexposed people from Singapore (Le Bert et al., 2020), the USA (Mateus et al., 2020) and Germany (Nelde et al., 2020; Schulien et al., 2020) to endemic HCoV protein sequences (Fig. 2). Without setting any identity threshold to report protein identity, we found that 76.3–83.1% of the SARS-CoV-2 epitopes had no homology to the four endemic HCoV species individually. In addition, 97 of the 177 epitopes (54.8%) had no homology to the proteome of all four endemic HCoVs combined (henceforth ‘unexplained’ epitopes). To investigate the potential source of ‘unexplained’ epitopes within the *Coronaviridae* further, we calculated the proportion of the 97 ‘unexplained’ epitopes with any homology to the proteome of each remaining coronavirus in our dataset (excluding SARS-CoV-2) (Fig. S2). The results suggest that a large proportion of ‘unexplained’ epitopes have homology to at least some of the *Betacoronaviruses* including SARS-CoV-1 and SARS-CoV-1-like coronaviruses within the Sarbecovirus sub-group.

**Fig. 3:**
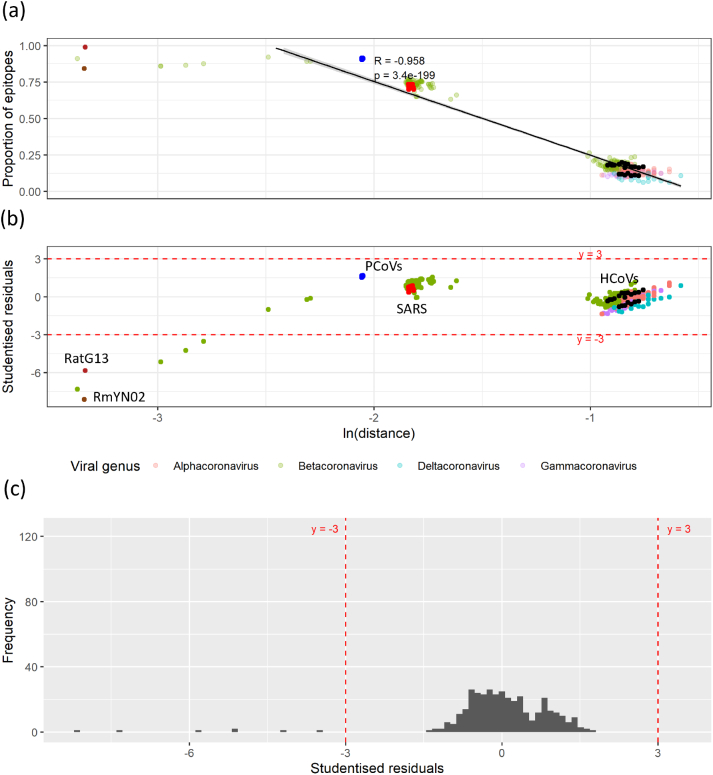
Relationship between the proportion of unexposed epitopes that have detectable sequence homology and the Mash distance to SARS-CoV-2 in a representative subset of the *Coronaviridae*. (a) Scatter plot and least squares regression line providing the proportion of epitopes with detectable homology to a coronavirus species (y-axis) and the natural logarithm of Mash distance to SARS-CoV-2 (x-axis). The dataset was filtered to only include 365 coronaviruses encompassing all unique host species, viral species and Mash distance combinations. These coronaviruses are coloured by viral genera with key members highlighted.

Additionally, given the overrepresentation of some coronavirus species within the dataset, we randomly subset the 2572 viral records to include only representative of each host and viral species that have non-identical Mash distances to the SARS-CoV-2 NCBI reference genome (Wuhan-Hu-1; NC_045512.2). Using the resultant 365 records, we found that the proportion of published epitopes with any homology to coronaviruses is strongly correlated with the natural logarithm of alignment-free Mash distance between the entire genome of each coronavirus relative to SARS-CoV-2 (Pearson's *r* = −0.958, *p* < 0.0001) (Fig. 3a). In fact, none of the 365 viruses in this filtered dataset had studentised residuals exceeding three, indicating that no coronaviruses within the dataset have homology to a significantly higher number of epitopes than expected by chance (Fig. 3b).

### 3.3 Possible sources for T-cell cross-reactivity beyond coronaviruses

To identify possible sources for the T-cell cross-reactivity observed in people unexposed to SARS-CoV-2, we also performed a protein BLAST search for all 177 experimentally validated epitopes against the NCBI non-redundant protein database (excluding the taxon *Coronaviridae*), storing the first 1000 hits in each case. A fraction of the epitopes (10/177) share partial homology with proteins from a very diverse range of taxa, including viruses, bacteria and unicellular eukaryotes (Table S2). However, the lowest Expect (E) value of the protein BLAST hits, which represents the number of similar hits expected by chance given the size of the database used and the length of the query (Tatusova and Madden, 1999), is 7.5. This suggests that all the hits shown in Table S2 could be explained by chance alone. Together with the wide diversity of taxa identified, the results suggest that there is no single candidate for the source(s) of the T-cell cross-reactive repertoire beyond the *Coronaviridae*.

## 4 Discussion

SARS-CoV-2 cross-reactive T-cells in healthy unexposed individuals have been identified as potentially important contributors to the immunological response to COVID-19. Prior exposure to globally circulating endemic coronaviruses present some of the strongest candidates for eliciting such cross-immunity. Though, the relative contribution of these coronaviruses to the reactive T-cell epitopes identified in multiple cohorts of healthy individuals have been only partially explored. We characterised the amino acid homology of SARS-CoV-2-derived T-cell epitopes reported in COVID-19 unexposed individuals from Singapore (Le Bert et al., 2020), the USA (Mateus et al., 2020) and Germany (Nelde et al., 2020; Schulien et al., 2020) against the entire proteome of the *Coronaviridae* family, including all major mammalian and avian lineages.

Following a comprehensive screen, we found that 54.8% of reported T-cell epitopes had no homology to the four human endemic coronavirus species (HKU1, OC43, NL63 and 229E) (Fig. 2), despite HCoV infections circulating widely in global human populations (Su et al., 2016). We note that the highest conservation to confirmed T-cell epitopes tended to be within members of the *Sarbecovirus* sub-group, which includes SARS-CoV-1, SARS-CoV-2, and a few related species that have been isolated mostly from bats and pangolins but are not known to have been in widespread circulation in humans. However, this homology can be well explained by the core phylogenetic relatedness of these viral species to SARS-CoV-2 (Fig. 3). Furthermore, SARS-CoV-2 infection leads to a heterogenous pattern of cell-mediated immune responses over the entire SARS-CoV-2 genome, largely falling outside of the spike protein, not enriched in the terminal end of ORF1ab largely conserved among the coronaviruses, and does not consistently lead to cross-reactivity with endemic HCoVs (Ferretti et al., 2020).

Our work adds to a growing suite of evidence that prior HCoV infections are not the only candidates responsible for cross-reactive T-cell epitopes in SARS-CoV-2 unexposed individuals. We argue that previous studies that presented empirical evidence of T-cell cross-reactivity with HCoV-derived peptides did not take into account the genetic relatedness of endemic HCoVs to SARS-CoV-2, placing an over-emphasis on these viruses as the source of pre-existing T-cell immunity. This opens the question as to what other antigens may have primed the intrinsic cross-reactivity identified (Campion et al., 2014) in pre-pandemic samples. A sizeable fraction of cross-reactive T-cell epitopes remains unexplained by prior exposure to any known coronavirus in circulation. It feels fairly implausible that the ‘unexplained’ cross-reactive epitopes are due to prior exposure to a yet undescribed coronavirus. Indeed, such a hypothetical yet-to-be described coronavirus would have needed to be in circulation globally until very recently and then vanished, which seems highly unlikely. Additionally, since we incorporated the whole known genetic diversity of coronaviruses in our analyses, which has been reasonably well sampled, such an unknown pathogen would likely have to be phylogenetically unrelated to any coronavirus characterised to date. As such, an unknown coronavirus would be an unlikely candidate for a source of this ‘unexplained’ T-cell cross-reactivity.

Possible alternative agents for the unexplained cross-reactive epitopes may include widespread microbes, or widely administrated vaccines. The tuberculosis bacille Calmette-Guerin (BCG) vaccines have been suggested as candidates providing some cross-immunity against SARS-CoV-2 (Tomita et al., 2020; Escobar et al., 2020). However, our screen of all 177 published T-cell epitopes found no homology to any *Mycobacterium* species (Table S2). As such, the evidence that BCG vaccination is a contributor to the T-cell cross-reactivity observed remains unconvincing. Instead we identify a diverse spread of putative antigens with low detectable homology. The presence of such a broad pre-existing repertoire of CD4^+^ reactive T-cells in healthy adults has previously been observed in the context of cross-reactivity to HIV and influenza infection, and interpreted as the result of prior exposure to environmental antigens (Su et al., 2013) or proteins in the human microbiome (Campion et al., 2014). It has also been postulated that the cross-reactive profile may take on an increasing role with age and immunological experience (Woodland and Blackman, 2006) which may result in high levels of inter-individual variation based on infection history and HLA type.

Admittedly, sequence homology is an indirect proxy for probing the source of T-cell cross-reactivity. Yin and Mariuzza (Yin and Mariuzza, 2009) reviewed five putative mechanisms of T-cell cross-reactivity, all of which highlight the complex and diverse molecular interactions of peptide, major histocompatibility complex (MHC) and T-cell receptors. In particular, molecular mimicry would suggest that conservation of structure can compensate for lower sequence homology (Macdonald et al., 2009; Wucherpfennig and Strominger, 1995; Quaratino et al., 1995). Deconvolving the relationship between sequence homology and cross-reactivity is evidently non-trivial and remains a limitation of our work. Indeed, we do not rule out the possibility that peptides of lower homology from members of the *Coronaviridae* can result in cross-reactivity. However, it remains evident that a high sequence homology improves the likelihood that structural or chemical characteristics are conserved, with empirical evidence that this is the case. For instance Mateus et al. (Mateus et al., 2020) found that only 1% of SARS-CoV-2:HCoV peptide pairs sharing 33–40% sequence homology were cross-reactive. Meanwhile, 21% of peptide pairs with 47–60% homology and 57% of peptides with >67% homology were cross-reactive. These findings highlight a positive association of sequence homology and the frequency of cross-reactivity, providing strong empirical evidence for our assumption that sequence homology is a good measure for inferring the source of T-cell cross-reactivity. Additionally, Grifoni et al. (Grifoni et al., 2020b) showed that 12 of 17 SARS-CoV-2 peptides with >90% sequence homology to experimentally-validated SARS-CoV epitopes were predicted to elicit a T-cell response. The authors then conclude that these peptides have a high probability of triggering a T-cell response, and could generate responses that are “cross-protective” across *Betacoronaviruses*. This serves as a precedent for using sequence homology to infer T-cell cross-reactivity. Finally, while a sequence homology-based approach may not be able to account for cross-reactivity as a result of structural homology, it offers scalability in that we can screen all known coronaviruses to date, which would not be feasible experimentally.

In conclusion, our results highlight the importance of considering the wider phylogenetic context of circulating antigens contributing to immunological memory to novel pathogens. The widespread and repeated exposure of global human populations to circulating endemic HCoVs is expected to have left an immunological legacy which may modulate COVID-19 pathogenesis. However, our results suggest that the extensive T-cell cross-reactivity previously reported cannot be solely explained by prior exposure to any known coronavirus in global circulation. It is nonetheless clear that the potential cross-reactive repertoire is widespread and present in cohorts of healthy people from multiple countries around the globe (Mateus et al., 2020; Grifoni et al., 2020a; Le Bert et al., 2020; Nelde et al., 2020; Braun et al., 2020; Peng et al., 2020; Schulien et al., 2020; Bacher et al., 2020; Sekine et al., 2020; Weiskopf et al., 2020b), even if perhaps at low avidity (Bacher et al., 2020). It remains to be established to what extent such cross-reactivity translates into immunity to SARS-CoV-2, both in terms of susceptibility to infection and symptom severity upon infection.

## Data and code availability

All source code used for the analyses can be found on GitHub (https://github.com/cednotsed/tcell_cross_reactivity_covid.git). Genomic data for the *Coronaviridae* were obtained from publicly available accessions on NCBI Virus. Twelve further bat and pangolin associated coronaviruses were also included downloaded from the GISAID repository, with full acknowledgements provided in Table S3. The list of epitopes used and the frequency table of CD4^+^ and CD8^+^ T-cell epitopes stratified by study cohort can be found in Table S4a and b respectively.

The following are the supplementary data related to this article.Supplementary Fig. S1 Conservation analysis of SARS-CoV-2-derived 15-mer peptides across the *Coronaviridae*. Maximum likelihood phylogeny and heatmap visualising the homology of SARS-CoV-2-derived 15-mer peptide sequences across the family, similar to that shown in Fig. 1 but using (a) 66% and (b) 80% as the protein BLAST homology threshold. Supplementary Fig. S1

Supplementary Fig. S2 Proportion of ‘unexplained’ epitopes that have any sequence homology to members of Coronaviridae. Raincloud plot (Allen et al., 2019) of the proportion of ‘unexplained’ epitopes that have detectable homology to each of the 2572 coronaviruses in our dataset (excluding SARS-CoV-2). Supplementary Fig. S2

Supplementary Table S1 Curated metadata of the 2572 viral records in the *Coronaviridae*. Supplementary Table S1

Supplementary Table S2 Protein BLAST results of 177 published epitopes against non-*Coronaviridae* proteins. Merged protein BLAST output of eight searches (https://tinyurl.com/y22o4t9z). Merging was performed using a custom *R* script. Supplementary Table S2

Supplementary Table S3 GISAID acknowledgements table for the 12 bat and pangolin coronavirus sequences. Supplementary Table S3

Supplementary Table S4 (a) List of 177 epitopes used in this study, including their respective study source and T-cell response type. (b) Frequency table generated from Table S4a stratified by study name and T-cell response type. Supplementary Table S4

## Acknowledgements and funding

L.v.D and F.B. acknowledge financial support from the 10.13039/100010897Newton Fund UK-China NSFC initiative (grant MR/P007597/1) and the 10.13039/501100000268BBSRC (equipment grant BB/R01356X/1). L.v.D. is supported by a UCL Excellence Fellowship. C.O. is funded by a NERC-DTP studentship. Finally, we acknowledge the large number of research groups openly sharing SARS-CoV-2 genomic and immunological data with the research community.

## Declaration of Competing Interest

A.B. is a cofounder of Lion TCR, a biotechnology company that develops T-cell receptors for the treatment of virus-related diseases and cancers but was not deemed to have any competing interests. The other authors have no competing interests to declare.
