# Supplementary material for: Pre-existing T cell-mediated cross-reactivity to SARS-CoV-2 cannot solely be explained by prior exposure to endemic human coronaviruses
Source: Infect Genet Evol. 2021 Nov;95:105075. doi: 10.1016/j.meegid.2021.105075 (PMC8428999; doi:10.1016/j.meegid.2021.105075)
Supplement: The following are the supplementary data related to this article.Supplementary Fig. S1 — Conservation analysis of SARS-CoV-2-derived 15-mer peptides across the Coronaviridae. Maximum likelihood phylogeny and heatmap visualising the homology of SARS-CoV-2-derived 15-mer peptide sequences across the family, similar to that shown in Fig. 1 but using (a) 66% and (b) 80% as the protein BLAST homology threshold. [file mmc1.docx]

Supplementary Materials


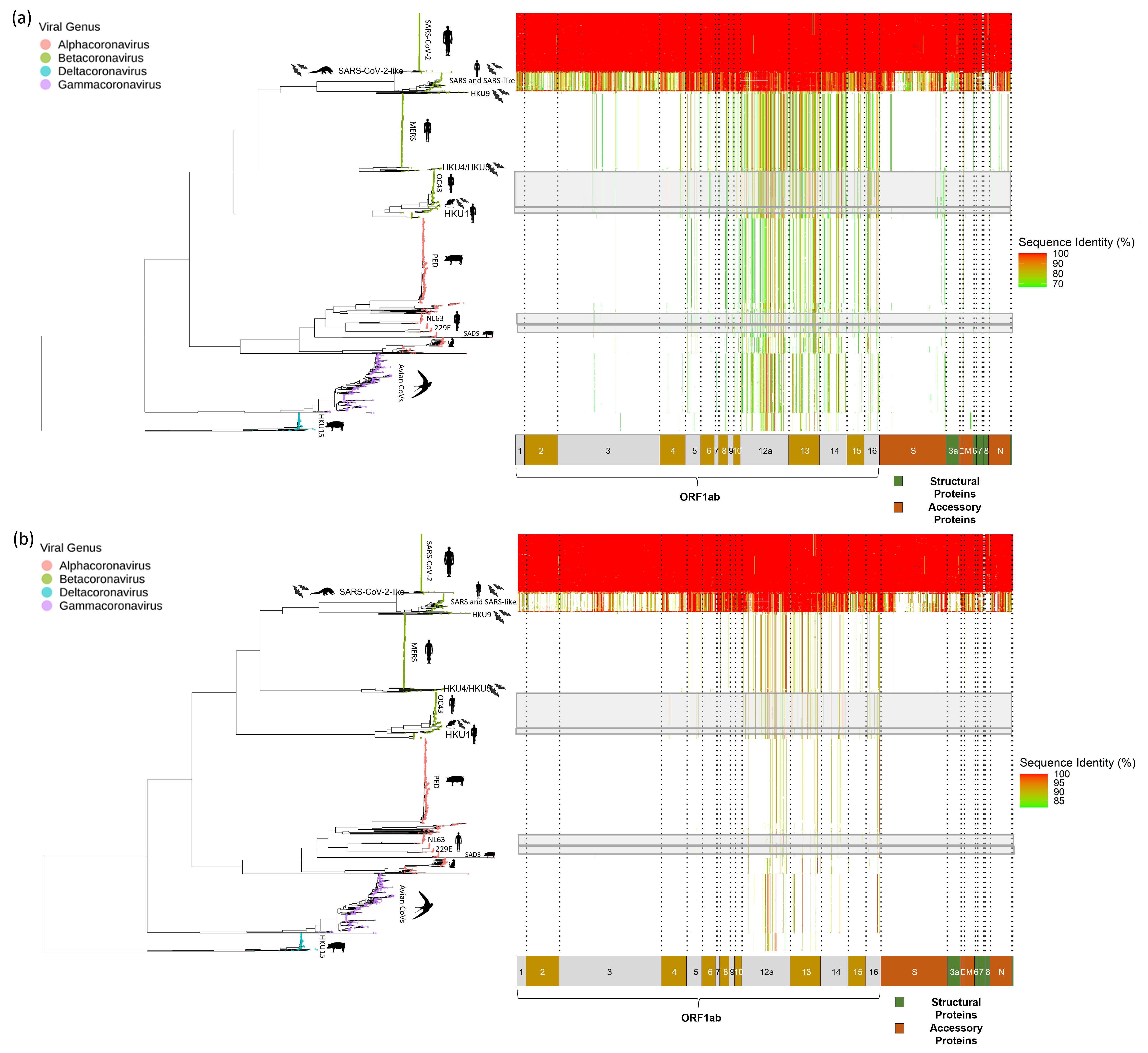


**Figure S1. Conservation analysis of SARS-CoV-2-derived 15-mer peptides across the *Coronaviridae*.** Maximum likelihood phylogeny and heatmap visualising the homology of SARS-CoV-2-derived 15-mer peptide sequences across the family, similar to that shown in **Figure 1** but using (a) 66% and (b) 80% as the protein BLAST homology threshold.
