# Supplementary material for: Pre-existing T cell-mediated cross-reactivity to SARS-CoV-2 cannot solely be explained by prior exposure to endemic human coronaviruses
Source: Infect Genet Evol. 2021 Nov;95:105075. doi: 10.1016/j.meegid.2021.105075 (PMC8428999; doi:10.1016/j.meegid.2021.105075)
Supplement: Supplementary Fig. S2 — Proportion of ‘unexplained’ epitopes that have any sequence homology to members of Coronaviridae. Raincloud plot (Allen et al., 2019) of the proportion of ‘unexplained’ epitopes that have detectable homology to each of the 2572 coronaviruses in our dataset (excluding SARS-CoV-2). [file mmc2.docx]

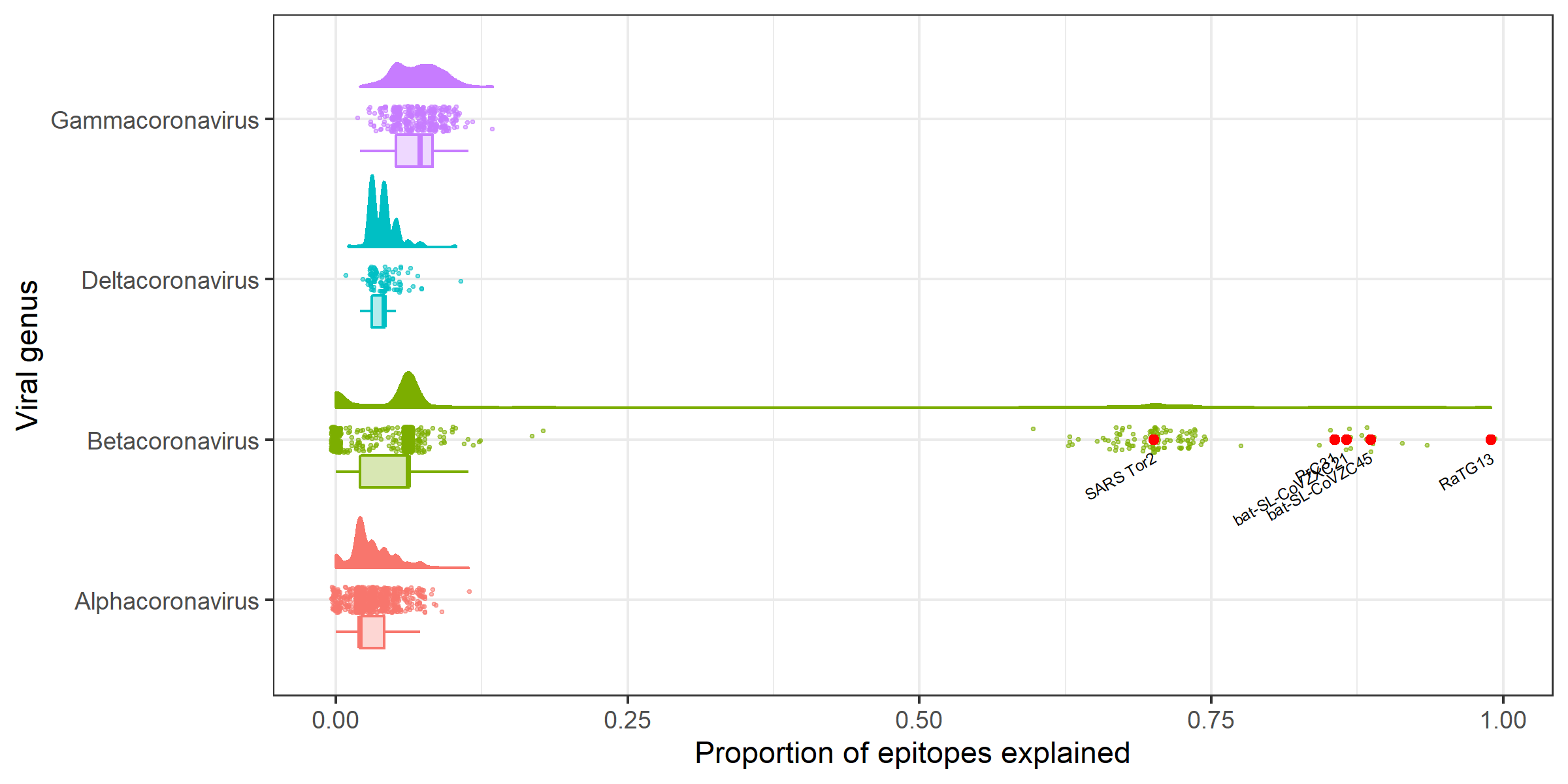


**Figure S2. Proportion of ‘unexplained’ epitopes that have any sequence homology to members of Coronaviridae.** Raincloud plot (63) of the proportion of ‘unexplained’ epitopes that have detectable homology to each of the 2572 coronaviruses in our dataset (excluding SARS-CoV-2).
