# Supplementary material for: Pre-existing T cell-mediated cross-reactivity to SARS-CoV-2 cannot solely be explained by prior exposure to endemic human coronaviruses
Source: Infect Genet Evol. 2021 Nov;95:105075. doi: 10.1016/j.meegid.2021.105075 (PMC8428999; doi:10.1016/j.meegid.2021.105075)
Supplement: Supplementary Table S3 — GISAID acknowledgements table for the 12 bat and pangolin coronavirus sequences. [file mmc5.pdf]

We gratefully acknowledge the following Authors from the Originating laboratories responsible for obtaining the specimens, as well as the Submitting laboratories where the genome data were generated and shared via GISAID, on which this research is based.

All Submitters of data may be contacted directly via www.gisaid.org

Authors are sorted alphabetically.

| Accession ID | Originating Laboratory | Submitting Laboratory | Authors |
|---|---|---|---|
| EPI_ISL_412977 | Shandong First Medical University & Shandong Academy of Medical Sciences | Institute of Microbiology, Chinese Academy of Sciences | Alice Catherine Hughes; Hong Zhou; Juan Li; Tao Hu; Weifeng Shi; Xing Chen; Yuhai Bi |
| EPI_ISL_1699443, EPI_ISL_1699444, EPI_ISL_1699445, EPI_ISL_1699446, EPI_ISL_1699447, EPI_ISL_1699448, EPI_ISL_1699449 | | | |
| see above | Shandong First Medical University & Shandong Academy of Medical Sciences | Shandong First Medical University & Shandong Academy of Medical Sciences | Alice C. Hughes; Edward C. Holmes; Hong Zhou; Jingkai Ji; Juan Li; Tao Hu; Weifeng Shi; Xing Chen; Yanhua Chen; Yuhai Bi |
| EPI_ISL_410721 | South China Agricultural University | South China Agricultural University | Lihua Xiao; Wu Chen; Yongyi Shen |
| EPI_ISL_852604, EPI_ISL_852605 | Virology Unit, Institut Pasteur du Cambodge | G5 Evolutionary Genomics of RNA viruses, Virology Department, Institut Pasteur | Alexandre Hassanin; Artem Baidaliuk; Christine Johnson; Deborah Delaune; Erik A Karlsson; Etienne Simon-Lorière; Fabiana Gámbaro; Jonna Mazet; Lucy Keatts; Ou Tey Putita; Philippe Buchy; Philippe Dussart; Tracey Goldstein; Veasna Duong; Vibol Hul; Vuong Tan Tu |
| EPI_ISL_1098866 | Yunnan Tropical and Subtropical Animal Viral Disease Laboratory, Yunnan Animal Science and Veterinary Institute, Kunming, Yunnan province, China; | Diarrhea department, National Institute for Viral Disease Control and Prevention, China CDC | Lili Li |
